# A Qualitative Exploration of Social Support in Males and Females Experiencing Issues With Infertility

**DOI:** 10.7759/cureus.29763

**Published:** 2022-09-29

**Authors:** Maya Pinzon, Shawna Rotoli

**Affiliations:** 1 Department of Molecular Biology, Rowan University School of Osteopathic Medicine, Stratford, USA

**Keywords:** psychosocial, gender differences, qualitative, infertility experiences, perceived social support

## Abstract

Introduction: The purpose of this study was to qualitatively investigate and compare male and female experiences of infertility in the context of social support.

Methods: A Qualtrics survey (Qualtrics, Provo, Utah, United States) was posted to online fertility support groups and the responses were thematically analyzed. Only participants that completed the qualitative component of the survey were included in the study. Responses were subsequently thematically analyzed.

Results: A sample of 110 participants (13 males and 97 females) were included in the present study. Thematic analyses revealed that isolation and loneliness, stigma, sentiments of misunderstanding, insensitive reactions, and others’ unhelpful attempts at support were general recurring themes, especially amongst females. Males predominantly reported negative emotional experiences and stigma, often feeling overlooked when compared with women despite being equally affected by these issues.

Conclusion: Our study provides insight into the factors that contribute to perceived isolation amongst the infertile population. These largely stemmed from feeling misunderstood and from others’ inexperience with infertility. Spreading awareness and facilitating dialogue and education across not only the infertile population but also the community, is therefore critical to begin addressing the mental health effects of infertility.

## Introduction

Infertility affects 15% of reproductive-age couples globally, with a continuously increasing prevalence [1] The experience of infertility has been shown to be both physically and psychologically stressful across all cultures and societies [2]. In fact, the experience of infertility is conditioned by social structure realities and as such, the current literature suggests that infertility is best understood as a socially constructed process rather than a merely physical condition [3].

Much of the present literature is focused on understanding which infertility, male or female is "worse,” rather than looking at ways in which their experiences may differ and why. Moreover, studies have primarily focused on comparing distress in infertile individuals with that of the general population, rather than focusing on how infertile individuals experience their day-to-day lives and what factors may be contributing to their distress [4].

The purpose of this study was to investigate the male and female experience of infertility in the context of a social system. Rather than looking at the relative effects of infertility compared with the general population, our study aims to explore the absolute effects and experiences of infertility on the individual.

## Materials and methods

Participants and study design

Participants were recruited through an anonymous Qualtrics survey (Qualtrics, Provo, Utah, United States) posted to online fertility support groups which consisted of individuals from across the United States. Participation was voluntary with no associated cost nor compensation, and all individuals above 18 years of age with reported current or prior issues with infertility were eligible to participate. This study was approved by the Rowan University Institutional Review Board with the approval number #Pro2020001151 (16 September 2021).

Survey tool

Participants completed a 24-question survey which included questions on demographics and infertility and support as measured by the Multidimensional Scale of Perceived Social Support (MSPSS). There was also a free response component consisting of one question. So as not to bias the participants, this question simply asked if there was anything they wished to share about their experience with infertility. The focus of this study is the free response component and thus only individuals who completed this section were included in our analysis. A thematic analysis was conducted on the open-ended responses through repetitive data reading and review, data coding, and subsequent theme identification.

## Results

Hundred and ten participants shared their experiences with infertility through an unstructured open-ended component of a larger survey. A total of 13 males and 97 females participated in this portion of the study with most respondents being White, between ages 30-35, married, heterosexual, with a bachelor’s degree as the highest level of education, and household income above $50,000 amongst both females and females. Demographic data are summarized in Table 1.

**Table 1:** Demographic characteristics of respondents (n= 110)

	Demographic Variables	n =110 (%)	Male sex at birth (n=13) (%)	Female sex at birth (n=97) (%)
Sex at birth	Male	13 (11.82)		
	Female	97 (88.18)		
Age range	18-23	1 (0.91)	1 (7.69)	0
	24-29	26 (23.64)	4 (30.77)	22 (22.68)
	30-35	54 (49.09)	3 (23.08)	51 (52.58)
	36-41	23 (20.91)	2 (15.38)	21 (21.65)
	42-47	6 (5.45)	3 (23.08)	3 (3.09)
Race/ethnicity	White	98 (89.09)	8 (61.54)	90 (92.78)
	Hispanic or Latino	7 (6.36)	4 (30.77)	3 (3.09)
	Black or African American	1 (0.91)	0	1 (1.03)
	Asian or Pacific Islander	2 (1.82)	0	2 (2.06)
	Other	2 (1.82)	1 (7.69)	1 (1.03)
Marital status	Single	5 (4.55)	3 (23.08)	2 (2.06)
	Married	95 (86.36)	9 (69.23)	86 (88.66)
	Divorced	1 (0.91)	1 (7.69)	0
	In a relationship (non-married)	7 (6.36)	0	9 (9.28)
Sexual orientation	Heterosexual or straight	96 (87.27)	12 (92.31)	84 (86.60)
	Bisexual	9 (8.18)	1 (7.69)	8 (8.25)
	Homosexual	1 (0.91)	0	1 (1.03)
	Pansexual	3 (2.73)	0	3 (3.09)
Level of education	High school degree or equivalent	9 (8.18)	2 (15.38)	6 (6.19)
	Bachelor’s degree	46 (41.82)	4 (30.77)	42 (43.30)
	Master’s degree	33 (30.00)	3 (23.08)	30 (30.93)
	Doctorate degree	8 (7.27)	1 (7.69)	7 (7.22)
	Other	14 (12.73)	3 (23.08)	12 (12.37)
Household income	< $24,999	4 (3.64)	2 (15.38)	2 (2.06)
	$25,000-$49,999	10 (9.09)	1 (7.69)	9 (9.28)
	$50,000-$74,999	53 (48.18)	2 (15.38)	11 (6.19)
	$75,000-$99,999	13 (11.82)	0	13 (13.40)
	$100,000-$149,999	30 (27.27)	6 (46.15)	24 (24.74)
	> $150,000	40 (36.36)	2 (15.38)	38 (39.18)

A number of themes emerged through a thematic analysis of the qualitative components of the survey. One of the most common themes that arose was how individuals that have not experienced infertility, don’t and can’t truly understand the struggle. This sentiment was especially predominant amongst females, with several specifically listing their partners as not understanding. Several examples of these responses are listed below:

“I don't think you truly understand what infertility feels like until you go through it.”

“Friends and family do not truly understand, unless they have personally lived it, which limits their ability to help despite their best intentions.”

“People that haven’t experienced [infertility] can’t understand how pervasive it can be (even my partner).”

“My partner, as wonderful as he is, can’t understand what the big deal is.”

“My husband is wonderful, but I don’t think he really understands infertility and how it affects me.”

Another common theme reported by both sexes was an inability to open up about their struggles, with females commonly reporting others’ lack of personal experience and inability to understand what they’re going through as a major deterrent for sharing their concerns.

“Because nobody from my friends/ family experienced infertility, it was difficult to talk with them about it.”

“None of my friends have personally experienced infertility so without the knowledge it is difficult for them to know how to support me.”

“One of the hardest parts of seeking support has been having to teach every person I’ve told how to be supportive… people want to [provide assurance] that things will work out, and in the case of infertility, they really might not. People don’t know how to be supportive in that situation.”

While many respondents reported great benefit from therapists or counselors in instances where they did not know anyone who shared a similar experience, one participant noted her therapist’s lack of assurance as the primary reason why she did not find her sessions helpful.

“She can’t promise everything is going to be ok, no one can.”

Further, a significant proportion of respondents indicated that such a disconnect often resulted in others not knowing how to respond and/ or responding inappropriately with unhelpful or minimizing comments, which further discouraged disclosure.

“[It’s] difficult to talk about this because [people] mostly don’t know how to respond or support - they say, ‘just relax.’”

“[It’s] very difficult for those not familiar with the struggles of infertility to understand your perspective and many times their attempts to comfort you or speak to you about your struggles can make you feel even more isolated.”

“Many do not know how to react [and] end up being unhelpful or hurtful with comments like ‘you can adopt’ [or] ‘just relax.’ We regret telling anyone about any of the process.”

“Sometimes what they say/do is worse than not having someone to talk to at all.”

Several respondents reported viewing infertility as a personal issue and thus resorted to keeping it a private matter, with others reporting fears of being judged and feeling embarrassed, ashamed, and overwhelmed. 

“Normally [I] would talk to family/ friends about problems, but infertility feels too personal to me.”

“I have not told [others] as I already know their views on barren women.”

Males particularly emphasized these sentiments, with several participants highlighting the stigmatizing nature (of specifically male-factor infertility) which further exacerbated their feelings of inadequacy and hesitancy for disclosure. 

“[I] was put down for being infertile.”

“This crushed my soul and decimated whatever self-confidence I had left.”

“I’ve given up on dating because everyone always wants kids and it’s always a source of angst.”

“Male infertility carries higher stigma than female infertility - sharing with family or friends about fertility issues is not something most of us are comfortable with.”

Moreover, several males reported feeling overlooked and less regarded in their experiences with infertility as compared with females.

“Nobody cares about the sadness of males.”

“Although perhaps this is more difficult on my partner [female-factor infertility] than myself, I’ve never felt that others understand that this feels exactly like a personal issue to me.”

While many respondents found it difficult and decided against sharing their infertility concerns with others, this did not necessarily indicate that such disclosure was not needed or desired.

“I haven't gotten as much support from my friends because I haven't shared a need for it, even though one exists.”

Another common theme, and the most frequently recurring phrases across respondents, were “loneliness and isolation.” These feelings stemmed in part from others’ inability to understand what they were going through without having experienced infertility themselves, as well as from its resulting inappropriate and/ or minimizing reactions.

“Infertility is a really isolating experience. People try to make well-meaning comments, but they can be callous and hurtful. I've learned it's usually better to keep stuff to myself than to ask for support from others.”

“Infertility is incredibly painful and isolating. It has affected every facet of my day-to-day life. I’ve become overwhelmed by feelings of despair but don’t know how to reach out to anyone for support.”

“Most people I spoke with viewed infertility as not a big deal/something that was all in my head.”

“If time has passed, people think you’d have come to terms with it by now”

Some participants suggested that feelings of isolation were in part a reaction to others’ general discomfort surrounding the issue of infertility.

“Most people will just pretend like infertility doesn’t exist, even if I’ve talked to them about it”

“Infertility is isolating, people (even family) like to brush over difficult situations or ignore them.”

“Even people who would normally be your support system don’t want to talk about infertility - it’s lonely and isolating”

“I got a lack of support from friends [as they] feel uncomfortable with my grief.”

However, several respondents also mentioned feeling isolated and unsupported by doctors as well.

“It’s difficult to be constantly questioned and overwhelmed with information. To doctors who work in infertility, they deal with this all day, but each person is new, different, and scared.”

Even when surrounded by a strong support system or others who previously struggled with infertility, many respondents reported still feeling alone.

“Even those who struggled with infertility still struggle to support others who go through it because it’s extremely personal and everyone takes it differently.”

“The journey is really hard and can feel isolating even when you are surrounded by people who love you” … “a spouse [often] struggle[s] to help because they’re experiencing their own pain.”

Further, several respondents reported a weakening in their overall relationships as a result of their struggles.

“Living with infertility will push your relationship with your partner to the brink. It has shaken our relationship to its core and in my opinion weakened it. Both my wife and I have fertility issues, and I wouldn't wish this hell on anyone.”

Several respondents shared their insights into what healthcare providers did well, as well as suggestions on what could be improved/what providers can do better.

“My doctor doesn't assign infertility as either my husband or my factor. Even when we mention it's likely female factor infertility, they're quick to let us know it could be either. It feels very supportive and doesn't place blame on either partner.”

“I wish children conceived through in vitro fertilization (IVF) was shared more freely among women. When we began to consider IVF we were fairly young [and] my spouse and I only felt comfortably beginning IVF after hearing [of a cousin’s IVF success story].”

“Doctors who work in infertility, they deal with this all day. But [they must] realize each person is new and different and scared.”

## Discussion

It is widely known that infertility poses a significant psychological burden, with many studies finding feelings of alienation and loss of control to be especially common [4,5]. Our study specifically found the phrase “loneliness and/or isolation” to be a common finding, reported recurrently and openly, particularly among female respondents (20%). This was seen even when individuals were surrounded by a support system and people who cared for them. Our findings suggest several possible factors that may have contributed to this sentiment: others’ lack of personal experience with the issue, others’ discomfort with the issue in general, perceptions that infertility is too private to share, as well as others’ insensitive or unhelpful reactions and attempts to support.

The most common reported factor overall (16%) was about how individuals that have not experienced infertility don’t and can’t truly understand the struggle. This is consistent with prior research findings [6]. Even partners were commonly listed as those who were not understanding, which is in accordance with prior findings [7]. This disconnect contributed to an overall difficulty to be open about their struggles which in turn contributed to and/or amplified feelings of isolation. Further, a lack of experience and understanding often translated into ignorance about how to react and respond to those struggling with infertility, which often resulted in unhelpful and even hurtful comments [8]. Several respondents reported that “just relax” was a frequent response to their struggles, which felt belittling and misguided as far as its impact on infertile individuals. Many respondents felt that such reactions stemmed in part from stigma and not wanting to talk about infertility, and from others wanting to offer assurance of a good outcome, but since certainty is not characteristic of infertility struggles, these responses were just not helpful.

Feelings of isolation didn’t end with a lack of understanding, as there is significant complexity underlying such a deeply personal issue that differs from individual to individual. This was a major factor that contributed to some individuals always “feeling like an outsider,” even when they were part of infertility support groups, or around others with shared experiences. While many respondents reported marked benefit and support from individuals with shared experiences or therapists specializing in infertility, which is consistent with prior research [9,10], some still felt isolated as they would compare themselves with others and often worried about having “a worse/the worst” story.

While females most commonly reported inadequate or unhelpful attempts at support, male responses were more centered around a sense of being neglected, with their feelings being mostly overlooked [11]. Reviews of literature indicate that a large portion of research is focused predominantly on females which leaves the male perspective understudied and contributes to the long-standing belief that infertility takes a bigger toll on the female [11, 12]. Our findings suggest that males recognize this, which influences their attitude in response. Feeling neglected or misunderstood has severe effects on self-esteem, relationships, and desire for disclosure [13]. This form of learned helplessness may contribute to a self-reinforcing cycle, furthering their detachment from others. This creates the misperception that males are less affected, which in turn reinforces the belief that there is a need to redirect research focus to the female experience. Efforts should thus be made to focus on the unique experiences and emotions that males may, and often do, experience in the context of infertility [13].

Further, our findings suggest a common belief that there is an increased stigma surrounding males with male-factor infertility (MFI) [12]. This further highlights a need for community-level education as well as psychosocial support for males with MFI to reframe their perception of their infertility as not something to be ashamed of and not something that should be stigmatized [14].

Our findings overall underscore the importance of speaking more freely about infertility especially given its pervasiveness, not placing blame, and educating the community to facilitate understanding and eliminate misunderstandings that contribute to the stigma surrounding infertility. Spreading awareness and facilitating dialogue and education about this common issue is critical [10], not only amongst friends and relatives, but in the population and society, as children, or the lack thereof, are in some way or another something we all relate to/have in common [15].

Our study has several strengths. Using a qualitative approach with a free response component, we were able to obtain a more nuanced and fuller understanding of the experience of infertility as participants were not prompted with specific, structured leading or suggestive questions. which allowed us to obtain the least biased collection of the most pressing issues at the forefront of participants’ minds. Additionally, participants were not drawn from convenience samples from clinics, which allowed us to obtain responses from individuals struggling with infertility regardless of whether they were getting treatment. This allowed for a more accurate representation of the infertility experience in general, rather than just of the experience of infertility treatment. Our study however was limited by the small number of male participants that chose to respond to the free response question. Given their unique perspectives and struggles, it may be of benefit for future studies to investigate these findings with a larger sample of males. As research suggests that females tend to participate more in surveys than males [16], it may be advantageous to try a different approach with regard to study methods design.

## Conclusions

Thematic analysis revealed several recurring themes including isolation and loneliness, sentiments that others do not understand what they are going through, stigma, and unhelpful support from others. Feelings of loneliness and isolation largely stemmed from others’ inexperience with infertility, discomfort with the issue in general, their own perception that infertility is too private to share, and others’ insensitive responses and unhelpful attempts at support. While females predominantly reported others’ inadequate attempts to support, males reported mostly a sense of being overlooked. These feelings of neglect had severe negative effects on self-esteem, relationships, and desire for disclosure, resulting in a feedback loop of further isolation and alienation. Spreading awareness and facilitating dialogue and education across not only the infertile population but also the community is therefore critical to begin addressing the mental health effects of infertility.
